# Investigating serum free light chains in patients with common variable immunodeficiency disorder in compare with other immunodeficiency diseases

**DOI:** 10.1038/s41598-026-41057-6

**Published:** 2026-03-13

**Authors:** Mohammad Hassan Bemanian, Majid Khoshmirsafa, Mina Ahmadi, Mohamad Nabavi, Saba Arshi, Zahra Chavoshzadeh, Sima Shokri, Morteza Fallahpour, Negin Jafariaghdam, Fatemeh Afrashteh, Samane Asefi, Mehrnaz Mesdaghi, Samin Sharafian

**Affiliations:** 1 Department of Pediatrics, School of Medicine, Iran University of Medical Sciences, Tehran, Iran; 2 Immunology Research Center, Institute of Immunology and Infectious Diseases, Iran University of Medical Sciences, Tehran, Iran; 3 Department of immunology, school of medicine, Iran University of Medical Sciences, Tehran, Iran; 4 Department Allergy and Clinical Immunology, Shahid Beheshti University of Medical Sciences, Tehran, Iran; 5 Immunology and Allergy Department, Mofid Children’s Hospital, Shahid Beheshti University of Medical Science, Tehran, Iran

**Keywords:** Common variable immunodeficiency, Serum free light chain, Kappa light chain, Lambda light chain, Agammaglobulinemia, CVID, Biomarkers, Diseases, Immunology, Medical research

## Abstract

Serum free light chains (sFLCs) have recently been introduced as a diagnostic biomarker in common variable immunodeficiency (CVID) patients. Most patients with CVID have undetectable or lower levels of sFLCs compared to people with other types of immune system deficiencies with hypogammaglobulinemia, except for patients with agammaglobulinemia (AGG). Decreased production of kappa (κ) and lambda (λ) light chains over immunoglobulins (Ig) suggests a malfunction in early B cell development or plasma cell dysfunction may cause CVID.

In this cross-sectional study, immunoturbidimetry was used to measure sFLCs in 70 immunodeficiency patients, including 39 patients with CVID, 31 patients with other immunodeficiencies with hypogammaglobulinemia such as combined immunodeficiency (CID), AGG, and other primary immunodeficiencies (PIDs), and 20 healthy controls. The levels of both κ and λ chains were significantly higher in patients with CID than patients with CVID and AGG patients (p < 0.05). The median (Q1–Q3) κ level in patients with other primary immunodeficiency disorders (PIDs) was significantly higher than that observed in patients with AGG and CVID (p < 0.01). Similarly, the median (Q1–Q3) λ level was higher in patients with other PIDs compared with those with AGG and CVID (p < 0.05).ROC analysis of κ and λ disclosed an area under the curve (AUC) for κ was 0.98 (95% CI: 0.96–0.99, *p* < 0.0001) and for λ was 0.95 (95% CI: 0.89–0.99, *p* < 0.0001) in patients with CVID. The sFLCs test can help distinguish between CVID and other immunodeficiency patients with hypogammaglobulinemia and the diagnosis of hypogammaglobulinemia.

**Supplementary Information:**

The online version contains supplementary material available at 10.1038/s41598-026-41057-6.

## Introduction

“Common variable immunodeficiency condition” (CVID) is the most prevalent symptomatic primary antibody deficiency (PAD), affecting between 1 case per 25,000 and 1 case per 50,000 individuals in Western countries^1^. This condition is diagnosed more frequently in adults, but it can also affect children. However, in pediatric cases, it is crucial not to label prematurely the disorder as CVID, since the underlying immunodeficiency is often more complex and may require further evaluation.

As reported by recent studies, adults in their third decade of life have a higher prevalence of the disease than children in their first decade of life. According to the diagnostic criteria established by the Pan-American Group for Immunodeficiency (PAGID) and the European Society for Immunodeficiencies (ESID), CVID is characterized by an onset of immunodeficiency after the age of four, absence of isohemagglutinins, poor antibody response to vaccines, and a marked reduction in serum levels of IgG and at least one other isotype—either IgA or IgM^2^.

In recent years, the widespread application of genome sequencing in patients with CVID has developed new opportunities for understanding the disease. The main aim is to identify the specific genetic determinants underlying the diverse phenotypes of CVID, thereby improving our knowledge of its pathogenesis. Investigations indicate that approximately 10–30% of CVID patients are affected by pathogenic monogenic disorders. Genes involved in immune regulation or critical stages of B cell biology, such as activation, survival, and maturation, are usually associated with these abnormalities^3,4^. Some genetic abnormalities identified in CVID include intrinsic B-cell defects such as CD19 deficiency, T-cell defects such as ICOS deficiency, mutations in TNF receptors, and rarer deficiencies involving MSH5, CD81, and CD20^5–8^ However, Nearly 70% of CVID cases, especially those with clinical complications, have a genetic basis that is unknown^9^.

The clinical spectrum for CVID may be extensive, but its main features are immune system dysregulation and intensified susceptibility to infections^10^. This background contributes to a wide range of comorbidities and a comprehensive set of diverse clinical and immunological characteristics, such as recurrent upper respiratory tract infections, bronchitis, pneumonia, sinusitis, and otitis^11^. The key laboratory feature of CVID is primary hypogammaglobulinemia. It is crucial to note that the diagnosis of CVID requires the exclusion of secondary causes of hypogammaglobulinemia, which is a fundamental step in the differential diagnosis. It’s important to rule out secondary causes before confirming CVID, as they may have a similar immunologic profile but require a different management approach. Primary hypogammaglobulinemia can originate through hyper-immunoglobulin M syndromes (HIGMS), deficits in IgG1, IgG2, and IgA, as well as ataxia telangiectasis (AT). Secondary hypogammaglobulinemia can occur when protein loss is high, like in cases of nephrotic syndrome, enteropathies, and burns, or when protein production is low, like in cancer, medications, and conditions that cause bone marrow failure. Several studies have recommended the use of clinical and immunological biomarkers to improve the diagnosis of this disease due to the uncertainty in genetic studies regarding the immunophenotype and clinical presentations^12–14^.

Immunoglobulins are made up of four polypeptide chains, comprising the heavy (H) and light (L) chains. There are two varieties of L chains, including κ and λ^15^. The presence of free light chains (FLCs) in serum is typically detected due to their approximately 40% overproduction compared to H chains. Serum free light chain (sFLC) nephelometry was introduced approximately two decades ago as a highly sensitive method for accurately quantifying circulating free light chains^16^. During B-cell maturation, excess light (L) chains are produced and stored in plasma cells, where they normally bond with heavy (H) chains. Surplus L chains that do not bind to H chains are released into the bloodstream as free light chains, so making their secretion is a useful indicator of B-cell activity^17,18^. In healthy individuals, the concentrations of free κ and λ chains are very low, about 3.3–19.4 mg/L for κ and 5.7–26.3 mg/L for λ. The normal κ/λ ratio ranges from 0.26 to 1.65, depending on the analytical method used^14^.

Variations in free light chain (FLC) levels have been observed among a range of immune-related conditions, including diabetes, cardiovascular diseases, multiple sclerosis, and various malignancies, as well as in inflammatory disorders such as hepatitis B and C infections, rheumatic diseases, and tick-borne illnesses^19^.

Currently, the serum FLC (sFLC) assay has emerged as a sensitive and reproducible marker of B-cell activity in primary B-cell disorders, particularly in primary antibody deficiencies (PAD) such as CVID^12^. Although sFLCs are highly specific, their sensitivity to distinguish CVID within the broader spectrum of PAD remains limited. It is becoming more apparent that sFLC measurements may have a prognostic value and could possibly inform therapeutic decision-making in individuals with CVID^20^. Only a few hereditary κ light-chain abnormalities have been comprehensively characterized since 1972.

In this study, we aimed to evaluate the diagnostic utility of sFLCs compared to other differential markers in CVID and to assess the potential positive correlation between CD27 expression and sFLC levels in affected patients.

## Materials and methods

### Patient selection

This cross-sectional study was performed between 2022 and 2023 at Rasoul Akram and Mofid Children’s Hospitals in Tehran, Iran. The study population consisted of two groups: patients with immunodeficiencies, especially those diagnosed with CVID, who were referred to the immunology clinic, and a cohort of healthy individuals. All immunocompromised participants were selected according to the ESID diagnostic criteria^2^.

The study protocol involving human blood samples was reviewed and approved by the Ethics Committee of Iran University of Medical Sciences (IUMS) with approval code IR.IUMS.REC.1401.396. As part of their routine management, selected patients received monthly intravenous immunoglobulin (IVIG) infusions. To ensure that IVIG administration did not affect FLCs measurements, blood samples were collected right before each infusion. All samples were stored at − 70 °C until analysis.

### Immunoturbidimetry

Following sample collection, serum free light chain (sFLC) concentrations were measured in all participants using the immunoturbidimetry method, following the manufacturer’s protocol (FREELITE, The Binding Site Group Ltd., Birmingham, United Kingdom). The reference intervals for κ and λ were considered 3.3–19.4 and 5.7–26.3 mg/L, respectively. Also, analytical sensitivity was 0.45 mg/l for both sFLCs.

### Statistical analysis

Statistical analyses were performed using SPSS v27, and data were reported as the medians and quartiles. The data’s normality was evaluated by the Kolmogorov-Smirnov test and based on whether the variables were parametric or non-parametric using either T-Test or Mann–Whitney U test. Also, the ROC (Receiver Operating Characteristic) curve, sensitivity, specificity, positive predictive value (PPV), and negative predictive value (NPV) were carried out to determine diagnostic indicators. All graphical representations were illustrated using GraphPad Prism v10.6. A *P*-value less than 0.05 was considered statistically significant.

## Results

### Characterization of patients

The study population consisted of 90 individuals, with 70 diagnosed with various immunodeficiency phenotypes, including 39 patients with CVID, 15 patients with combined immunodeficiency (CID), 11 patients with X-linked and autosomal recessive AGG, and 5 patients with other primary immunodeficiencies (PIDs), including 1 case of HIGMS, 2 cases of Ataxia–Telangiectasia (AT), and 2 cases of IgA deficiency, and also 20 healthy individuals as healthy controls (HCs). The clinical presentation and demographic characteristics are displayed in Table 1. All PID groups showed a 100% rate of infection, while patients with CVID (32%), CID (21.4%), and AGG (18.2%) were the most likely to have autoimmune manifestations. The occurrence of granulomatous disease is rare, with the highest frequency observed in CID (7.1%) and CVID (2.4%). Lymphoproliferative complications were found to be more prevalent in the CVID group (26.8%) than in CID (21.4%) and AGG (9.1%). Allergic manifestations were not common, with a range of 7–8% across CVID, CID, and AGG, and were absent in other PIDs. Overall, Infections and allergic diseases represented the most (90–100%) and least (0–8%) common clinical manifestations among patients with CVID, respectively. Patients with CVID had the highest mean age (33.6 ± 15.1), reflecting a later onset compared with CID, AGG, and especially other PIDs, which showed markedly younger age distributions. Of the 39 patients with CVID initially enrolled, 3 were excluded following further evaluation due to changes in their immunological profiles and clinical presentations that no longer met the diagnostic criteria for CVID.

**Table 1:** Clinical presentation and clinical characteristics (HCs = healthy controls, CVID = common variable immunodeficiency, CID = combined immunodeficiency, AGG = agammaglobulinemia, PID = primary immunodeficiencies, SD = standard deviation, Min: Minimum, Max: Maximum).

**Groups (n)**	**Gender: F/M (%)**	**Age**		**Infection (%)**	**Autoimmunity (%)**	**Granuloma (%)**	**Lymphoprolif** **-** **eration (%)**	**Allergy (%)**
		**Mean ± SD**	**Min-Max**					
HCs (20)	9/11 (45/55)	28.8±14.8	4-44	-	-	-	-	-
CVID (36) †	19/17 (53/47)	33.6±15.1	6-65	36 (100)	13 (32)	1 (2.4)	11 (26.8)	3 (7.3)
CID (15)	8/7 (53/47)	26.36±14.1	3-41	15 (100)	3 (21.4)	1 (7.1)	3 (21.4)	1 (7.1)
AGG (11)	3/8 (27/73)	26.82±7.8	13-37	11 (100)	2 (18.2)	0 (0)	1 (9.1)	2 (8.2)
Other PIDs (5)	2/3 (60/40)	15.60±8.1	8-29	5 (100)	0 (0)	0 (0)	0 (0)	0 (0)

† Three of the 39 CVID patients were excluded due to the phenotype deviation which is the specific diagnostic criteria targeted in this study.

### Serum free light chains levels

**Fig. 1:**
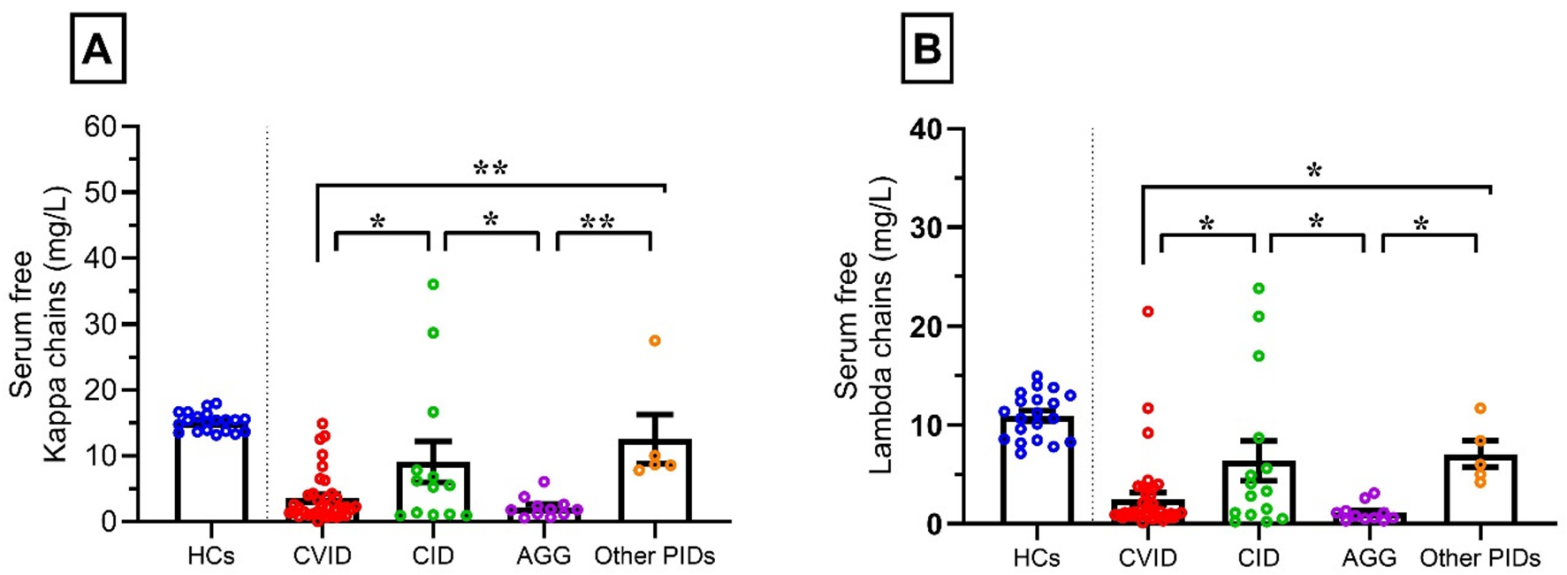
Serum free κ (**A**) and λ (**B**) chains distribution in different phenotypes of immunodeficiency Significance levels of comparison between groups: *P ≤ 0.05, **P ≤ 0.01. (κ = kappa, λ = lambda, CVID = common variable immunodeficiency, CID = combined immunodeficiency, AGG = agammaglobulinemia, PID = primary immunodeficiencies).

The median (Q1-Q3) of the κ chain was 2.1 (1.1–4.2) in patients with CVID, 5.5 (1.0–12.2.0.2) in patients with CID, and 1.8 (1.1–2.6) in patients with AGG. Also, the median (Q1-Q3) of the λ chain was 1.1 (0.8–3.8) in patients with CVID, 3.3 (0.9–8.7) in patients with CID, and 0.8 (0.5–1.3) in patients with AGG (Table 2). In accordance with the analytic assay, the Median (Q1-Q3) of both κ and λ chains in the HCs group was significantly higher than patients with CVID (*p* < 0.0001), patients with CID (*p* < 0.05), and patients with AGG (*p* < 0.0001). Additionally, the Median (Q1-Q3) of the κ and λ chains in patients with CID was higher than that in patients with CVID (*p* < 0.05) and patients with AGG (*p* < 0.05). The results demonstrated that the median (Q1–Q3) κ level in patients with other PIDs was significantly higher than that observed in patients with AGG and CVID (*p* < 0.01). Similarly, the median (Q1–Q3) λ level was higher in patients with other PIDs than pared with those with AGG and CVID (*p* < 0.05). (Fig. 1, Supplementary Table 1)

Furthermore, the Median (Q1-Q3) κ/λ ratio was calculated and found to be 1.95 for patients with CVID, 1.66 for patients with CID, 2.25 for patients with AGG, 1.45 for patients with other PID, and 1.40 for HCs, which presented non-significant difference in κ/λ ratio between the studied groups. However, the median (Q1-Q3) κ + λ, which was 3.2 (1.92–7.87) for patients with CVID, 7 (1.8–17) for patients with CID, 2.7 (2–3.1.1) for patients with AGG, 14.7^12–27,27^ for patients with other PID, and 25.92 (23.88–28.67) for HCs, showed a significant decrease in all patients compared with HCs (Table 2).

**Table 2:** Distribution of serum free κ and λ light chains (mg/L) among diagnostic groups (Q= Quartile, Min: Minimum, Max: Maximum, HCs = healthy controls, CVID = common variable immunodeficiency, CID = combined immunodeficiency, AGG = agammaglobulinemia, PID = primary immunodeficiencies).

	**Serum free light chain**	**HCs**	**CVID**	**CID**	**AGG**	**Other PIDs**
**Median (Q1-Q3)** **Min-Max**	Kappa	15.4 (13.7-16.2) 13.2-18.0	2.1 (1.1-4.2) 0.0 – 14.9	5.5 (1.0-12.2) 0.9 – 36.0	1.8 (1.1-2.6) 0.6 – 6.1	8.7 (8.4-14.4) 7.8 – 27.5
	Lambda	10.97 (8.5-12.9) 7.1 – 14.9	1.1 (0.8-3) 0.1 – 21.5	3.3 (0.9 – 8.7) 0.2 – 23.8	0.8 (0.5-1.3) 0.3 – 3.1	6 (4.8-9.2) 4.2 – 11.7
**κ/λ ratio**	Kappa /Lambda	1.40	1.95	1.66	2.25	1.45

### Correlation of sFLC values with CD27^+^IgD^−^IgM^−^ B cells and immunoglobulin serum levels

**Fig. 2:**
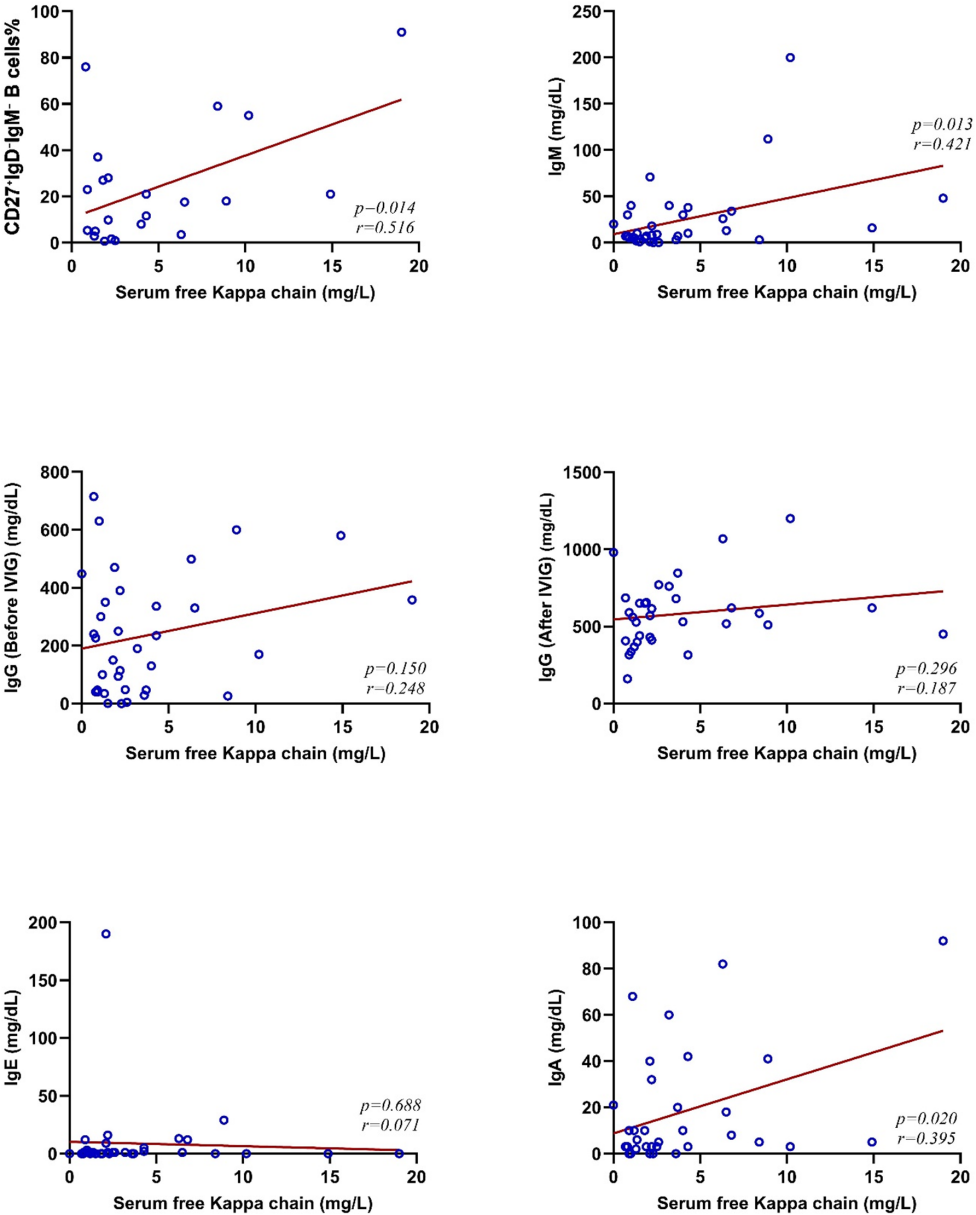
The correlation between serum free κ chain with the frequency of CD27+IgD-IgM- B cells and immunoglobulin serum levels in patients with CVID

**Fig. 3:**
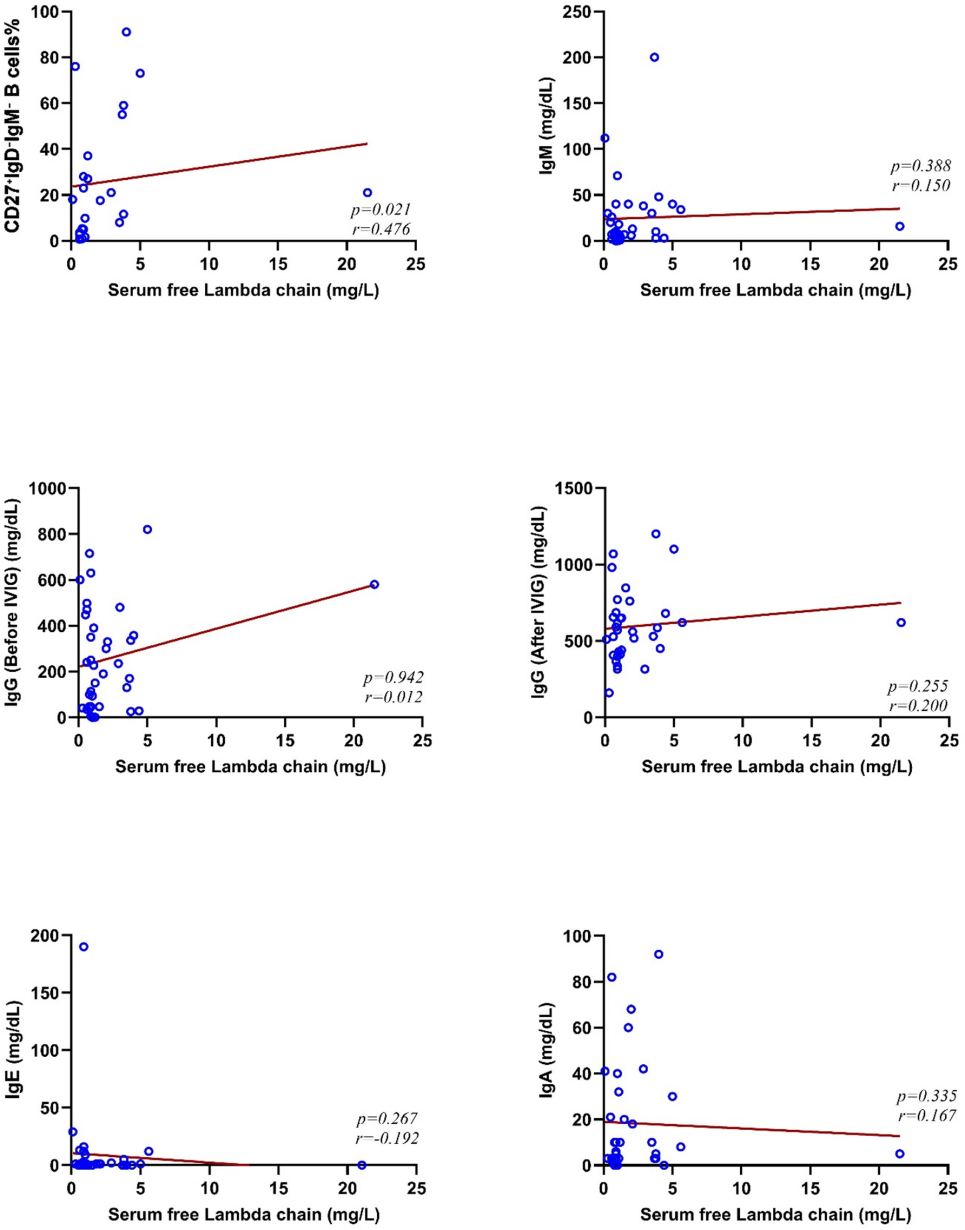
The correlation between serum free λ chain with the frequency of CD27+IgD-IgM- B cells and immunoglobulin serum levels in patients with CVID

As shown in Figs. 2 and 3, we observed a direct correlation between serum free κ and λ chains with the frequency of CD27^+^IgD^-^IgM^-^ B cells in CVID patients (*p* = 0.014, *r* = 0.51 for κ chain and *p* = 0.021, *r* = 0.476 for λ chain). Also, the association of immunoglobulin serum levels and CD27 + IgD-IgM- B cells showed a notable direct correlation between IgM (*p* = 0.013, *r* = 0.421) and IgA (*p* = 0.020, *r* = 0.395) and serum free κ chain level.

### Diagnostic indicators of sFLCs assay in CVID, CID and AGG patients

The results showed great sensitivity and specificity in the analysis of serum free κ and λ chains in CVID, CID, and AGG groups. The area under the curve (AUC) (95% CI) for serum free κ chain was 0.98 (0.96–0.99, *p* < 0.0001), 0.77 (0.55–0.99, *p* < 0.01), and 0.99 (*p* < 0.0001), respectively in CVID, CID, and AGG groups. Also, the AUC (95% CI) for serum free λ chain was 0.95 (0.89–0.99, *p* < 0.0001) in CVID, 0.78 (0.57–0.98, *p* < 0.01) in CID, and 0.99 (*p* < 0.0001) in AGG.

**Fig. 4:**
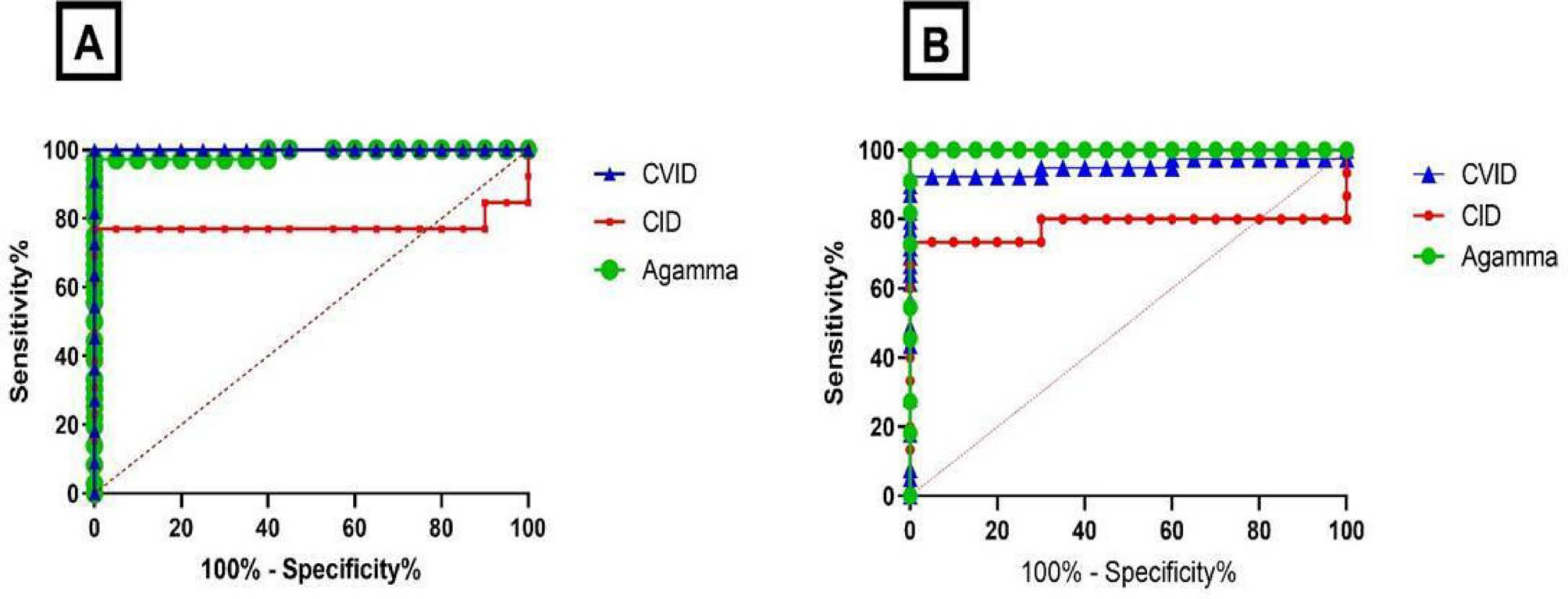
Assay of sensitivity and specificity of κ (**A**) and λ (**B**) in CVID, CID, AGG and HCs groups (sFLCs = serum free light chain, HCs = healthy controls, CVID = common variable immunodeficiency, CID = combined immunodeficiency, Agamma = agammaglobulinemia, PID = primary immunodeficiencies).

The cut-off value in sFLC was determined as Median − 3SD which was calculated as 10.86 and 3.96 in serum free κ and λ chains. The sFLC showed a great predictive value in patients with CVID and AGG, which was 91.67%, 100%, 100%, and 86.96% in Sensitivity, Specificity and positive predictive value (PPV), and negative predictive value (NPV) of serum free κ chain in CVID patients, respectively. Furthermore, Sensitivity, Specificity and PPV and NPV were presented as 87.18%, 100%, 100%, and 80%, respectively CVID patients in serum free λ chain (Fig. 4; Table 3).

**Table 3:** Analysis of sensitivity and specificity of sFLCs values in CVID, CID, AGG groups. (CVID = common variable immunodeficiency, CID = combined immunodeficiency, AGG = agammaglobulinemia, AUC = area under the curve, PPV = positive predictive value, NPV = negative predictive value).

	**CVID Kappa†**	**CVID** **Lambda†**	**CID** **Kappa**	**CID** **Lambda**	**AGG** **Kappa**	**AGG** **Lambda**
True positive	33	34	10	8	11	11
False negative	3	5	3	7	0	0
False positive	0	0	0	0	0	0
True negative	20	20	20	20	20	20
Sensitivity	91.67	87.18	76.92	53.33	100	100
Specificity	100	100	100	100	100	100
PPV	100	100	100	100	100	100
NPV	86.96	80	86.96	74.07	100	100
AUC	0.98	0.95	0.77	0.78	0.99	0.99
95 % Confidence interval	0.96-0.99	0.89-0.99	0.55-0.99	0.57-0.98	0.99	0.99
*p* - value	<0.0001	<0.0001	<0.01	<0.01	<0.0001	<0.0001

† The cut-off value in sFLC was determined as Median -3SD.

## Discussion

Early and precise diagnosis of CVID remains challenging due to its heterogeneous clinical and immunologic presentation. While recent cohort studies have improved our understanding of CVID, there are still unmet clinical needs and optional strategies to prevent long-term complications, including immune dysregulation and malignancy^21,22^. To guide appropriate management, it is crucial to perform early stratification and precise phenotyping at diagnosis from a clinical perspective^23^.

In this study, we evaluated serum free light chains in individuals with CVID, CID, AGG and other primary immunodeficiencies (PIDs) and compared them with healthy controls to determine the diagnostic contribution of sFLCs. The findings show sFLCs effectively differentiated performance in distinguishing PID patients from healthy individuals, with the highest diagnostic accuracy in AGG, where both κ and λ chains showed 100% sensitivity and specificity, achieved an AUC of 0.99. This outcome is consistent with the profound B-cell maturation defect in AGG, resulting in an absence of immunoglobulin production and consequently markedly reduced FLC synthesis.

In CVID, κ chains demonstrated slightly greater sensitivity than λ (91.67% vs. 87.18%), but both chains kept their specificity at 100%. Decreased sFLC levels are strongly predictive of CVID as indicated by the high PPV values; however, the lower NPV (86.96% for κ; 80% for λ) is due to the immunologic heterogeneity and variability in B-cell maturation pathways. In CID, sensitivity was moderate, particularly for λ (53.33%), though specificity remained 100%. This pattern suggests that while reduced sFLCs are highly specific, they are not sufficient as an independent screening tool for CID. The modest AUC values in CID (0.77–0.78) indicate that more broader biomarker combinations are required for this group.

Our results are in line with Guevara-Hoyer et al. (2020) findings, who reported significantly lower κ and λ levels in CVID compared with other PIDs^24^.

Unexpectedly, κ was elevated in 10 CVID patients and λ in four. Clinical follow-up showed autoimmune enteropathy in one patient treated with abatacept, B-cell lymphoma in another, and an ICOS mutation in a third patient, which led to reclassification as CID following confirmation of these findings.

This supports Scarpa et al. (2020)’ finding that elevated sFLCs may serve as a biomarker for autoimmunity or malignancy surveillance in CVID^25^. In contrast, although sFLC levels were uniformly reduced in AGG, the decrease did not reach statistical significance for distinguishing AGG from CVID.

We found a significant link between the frequency of CD27 + IgD-IgM-B-cells and κ/λ levels, which is consistent with Guevara-Hoyer et al.^26^. Given that The maturation of switched-memory B cells into plasma cells implies that reduced memory B cell output translates into diminished FLC production^27^. In CVID, κ and λ reductions reflect impaired B-cell differentiation; however, a correlation with IgG is not observed due to uniformly low IgG by diagnostic criteria. Instead, IgA and IgM variability across individuals offers greater correlation potential.

In terms of serum FLC levels, healthy participants displayed expected physiological FLC levels, whereas AGG patients showed the lowest κ and λ values (1.8 and 0.8). CID patients exhibited moderate reductions and CVID patients demonstrated a more selective κ reduction (2.1 vs. 1.1), suggesting differential disruption of light-chain synthesis. Additionally,s the κ/λ ratio increased in CVID and AGG and decreased in CID, mirroring differences in immunopathological profiles. Previous study by Grey et al. supports a κ/λ ratio of ~ 2:1 in AGG^28,29^, and Unsworth et al. proposed that abnormal κ/λ ratio in PAD reflects defective B-cell function rather than clonal expansion^12^. We suggest that κ/λ ratio changes or sudden sFLC elevation may serve as early warning indicators for malignancy and should prompt additional evaluation such as LDH and β2-microglobulin^26,27^.

This study has certain limitations that should be acknowledged. First, the relatively small sample size, particularly within the subgroup of patients with other primary immunodeficiencies (PIDs), may limit the statistical power of some comparisons and increase the risk of Type II errors. Second, the inherent heterogeneity of the analyzed population—encompassing diverse diagnostic entities under the broad categories of CVID, AGG, and other PIDs—could influence the observed biomarker profiles and affect the generalizability of our findings. While this reflects the real-world clinical spectrum, it may introduce confounding variables. Future studies with larger, more homogenous cohorts are warranted to validate these preliminary findings and to explore the diagnostic utility of immunoglobulin free light chains in specific, well-defined immunodeficiency subgrou.

Taken together, these findings support the use of sFLC measurement as a primary diagnostic tool in hypogammaglobulinemia and during clinical follow-up. To better understand FLC phenotypes specific to certain diseases, it is necessary to have larger prospective cohorts and standardized measurement techniques, such as ELISA, as variability in sFLC patterns reflects heterogeneity within CVID. Future research in immunology may refine the classification of CVID and allow the separation of presently grouped phenotypes into distinct groups.

## Supplementary Information

Below is the link to the electronic supplementary material.

Supplementary Material 1
